# Molecular diversity and high virulence of *Legionella pneumophila* strains isolated from biofilms developed within a warm spring of a thermal spa

**DOI:** 10.1186/1471-2180-13-17

**Published:** 2013-01-28

**Authors:** Zineddine Chaabna, Françoise Forey, Monique Reyrolle, Sophie Jarraud, Danièle Atlan, Dominique Fontvieille, Christophe Gilbert

**Affiliations:** 1 UMR CARRTEL, Université de Savoie-INRA, Le Bourget du Lac, F-73376, France; 2 Centre de Biologie et Pathologie Est, Centre National de Référence des Légionelles, Bron, F-69677, France; 3 Université Lyon, INSERM U851, Université Lyon 1, Lyon, F-69008, France; 4 Université de Lyon, CNRS UMR 5240, Université Lyon1, Villeurbanne, F-69622, France

**Keywords:** *Legionella pneumophila*, Biofilms, Warm spring, Biodiversity, Virulence

## Abstract

**Background:**

Several cases of legionellosis have been diagnosed in the same French thermal spa in 1986, 1994 and 1997. *L. pneumophila* serogroup 1 (Lp1) strains have been isolated from several patients, but the source of contamination was not identified despite the presence of different Lp1 in water samples of the three natural springs feeding the spa at this period.

**Results:**

Our strategy was to investigate *L. pneumophila* (Lp) strains from natural biofilms developed in a sulphur-rich warm spring of this contaminated site. Biofilm analysis revealed the presence of three Lp serogroups (Lp1, Lp10 and Lp12). Surprisingly, Lp10 and Lp12 were not reported in the previous described studies from water samples. Besides, the new seven Lp1 we isolated exhibit a high molecular diversity and have been differentiated in five classes according to their DNA genome patterns obtained by PFGE and *mip* sequences. It must be noted that these DNA patterns are original and unknown in databases. Interestingly, the 27 Lp environmental strains we isolated display a higher cytotoxicity and virulence towards the amoeba *Acanthamoeba castellanii* than those of known Lp1 epidemic strains.

**Conclusion:**

The characteristics of *Legionella pneumophila* Lp1 strains isolated from the warm spring are in agreement with their presence in biofilms and their probable long-term persistence in this ecosystem.

## Background

The human pathogen *Legionella pneumophila* causes a severe pneumonia so-called legionellosis or Legionnaires’ disease (LD); this Gram negative bacterium was identified after the 1976 Philadelphia outbreak during the American Legion convention where 29 people succumbed [1]. Further outbreaks were associated with aerosol-producing devices like showers, cooling towers, whirlpools and fountains, but Rowbotham was the first to show a link between *Legionella* ecology and LD [2,3]. Actually, *L. pneumophila* is ubiquitous in aquatic environment and is able to multiply intracellularly in fresh water protozoa.

*L. pneumophila* displays 15 serogroups but the majority of human cases are due to the serogroup1 (Lp1) (84% worldwide, 95% in Europe) [4,5]. Lp1 is frequently found in the environment and accounts for 28% of environmental isolates in France. Other *Legionella* species, as *L. anisa, L. dumoffii* and *L. feeleii* that frequently colonize the water distribution systems, are rarely involved in human disease [4]. These data suggest that the high frequence of LD involving Lp1 is not due to its predominance in the environment but rather linked to a higher virulence than other species or serogroups of *Legionella*. The only exception is *Legionella longbeachae* accounting for 30% of human cases in Australia and New-Zealand, and even 50% of cases in South Australia [6]. In contrast to *L. pneumophila, L. longbeachae* is found predominantly in potting soil and transmitted by inhalation of dust of contaminated soils.

A lot of attention has been paid to the identification of Lp1virulence factors. It is now recognized that the co-evolution between eukaryotic hosts and *L. pneumophila* had led to the selection of a set of virulence factors which allow this bacterium to exploit host cellular processes; among these factors, eukaryotic-like proteins, encoded by genes identified on the basis of genome sequence analysis, are involved in different steps of the *Legionella* intracellular cycle [5,7-10]. Recently, comparison of *Legionella* genome sequences has shown that some genes encoding the lipopolysaccharide biosynthesis were specific of Lp1 and constitute specific markers for the molecular typing [11].

We focused our attention on the identification and virulence capacities of different serogroups of *L. pneumophila* strains present in the French thermal spa where five cases of legionellosis were diagnosed in 1986, following by two cases in 1994 and 1997 [12,13]. In order to determine the source of infection, water samples had been collected throughout the water distribution system as well as the three natural springs (S, sulphur; A, alum and P, cold) and two bore holes feeding the system. Eighty one *L. pneumophila* strains belonging to five serogroups (27 Lp1, 1Lp2, 62 Lp3, 3 Lp6 and 9 Lp13) had been identified from water samples collected over a two-year period (1997–1998); thus this water system appeared mainly contaminated by Lp1 and Lp3, also present in two natural spring (S and A). Nevertheless, comparative analysis of genomic DNA, by PFGE (“Pulse Field Gel Electrophoresis”), of both clinical Lp1 isolated from patients and environmental Lp1 isolates did not allow identifying the source of infection.

In this study, our goal was to identify legionellae directly virulent towards protozoa and as a consequence with the ability to survive in a specific environment, like the spring S characterized by a temperature of 37°C and a high level of sulphates and thiosulphates as the calcium and sodium salts [12]. Thus, we isolated legionellae from natural biofilms developed on glass slides immersed in this contaminated spring. After typing by different approaches, the DNA genome diversity of these environmental Lp strains was analyzed, and their virulence and cytotoxicity towards the amoeba *Acanthamoeba castellanii* were compared to those of well-known French clinical isolates (Lp1 strains Lens, Paris and Lorraine).

## Results

### Phenotypic analyses and serotyping of environmental *L. pneumophila* isolates

*Legionella* cells developed in natural biofilms in the spring S were extracted according to the French AFNOR NFT90-431 procedure as described in Material and Methods. Independent bacterial colonies developed on GVPC in 5 days at 37°C were screened according to a fritted glass appearance with a green gleam. This assay provided us 5 isolates named LAXA from the campaign of August 2010 and 25 isolates named LAXB from December 2010 (Table 1). Then, serotyping was performed with two different latex agglutination tests (Oxoid kit and bioMérieux reagents) consisting of blue latex particles sensitized with specific rabbit antibodies of different serogroup (sg) antigens of *L. pneumophila*. These tests allowed us to show that all *Legionella* strains we isolated from biofilms belong to the specium *pneumophila* and to three sg (1, 10 and 12) with the following distribution: 7 Lp1, 5 Lp10 and 15 Lp12 (Table 1). At this stage, we were not able to characterize 3 strains by serotyping (LAXB11, LAXA53 and LAXA54). Besides, the 27 environmental positively serotyped Lp isolates also displayed an auxotrophy for cysteine, a known characteristic of legionellae (data not shown). Interestingly, analysis of the catalase activity of these 27 Lp strains allowed us to show that Lp*.* strains were characterized by a very weak production of gas bubbles (Cat^-^ phenotype) from the substrate H_2_O_2._ Actually, *L. pneumophila* displays two genes, *katA* and *katB,* encoding catalase-peroxidases, but functionaly theses enzymes exhibit only the peroxidatic activity [14,15]. By contrast *L. anisa* and *L. micdadei* (Table 1) and *E. coli* DH5α (data not shown) were able to generated rapidly numerous gas bubbles (Cat^++^ phenotype) and *L. longbeachae* and *L. taurinensis* were characterized by a clear gas emission but at a significantly lower rate than *L. anisa*. Thus, the Cat^-^ phenotype can be considered as a good tool for screening *L. pneumophila* strains among *Legionella* genus [16].

**Table 1:** **Typing of 30 environmental *Legionella *isolates from biofilms developed in the contaminated S spring**

			**Serotyping**		**Molecular typing**					
**Campaign**	**Environm. isolates**	**Catalase activity**	**Oxoid kit**	**bioMérieux reagents**	***16S rRNA***	***mip***	***lpg 1905***	***lpg 0774***	***wzm***	**Typing**
**LAXB**	1	-	Lp2-14	Lp10	+	+	+	-	-	Lp
	2	-	Lp2-14	Lp12	+	+	+	-	-	Lp
	3	-	Lp2-14	Lp10	+	+	+	-	-	Lp
	4	-	Lp2-14	Lp12	+	+	+	-	-	Lp
	5	-	Lp2-14	Lp12	+	+	+	-	-	Lp
	6	-	Lp1	Lp1	+	+	+	+	+	Lp1
	7	-	Lp2-14	Lp12	+	+	+	-	-	Lp
	8	-	Lp1	Lp1	+	+	+	+	+	Lp1
	9	-	Lp2-14	Lp12	+	+	+	-	-	Lp
	10	-	Lp2-14	Lp12	+	+	+	-	-	Lp
	11	-	NSR*	NSR*	+	+	+	-	-	Lp
	12	-	Lp1	Lp1	+	+	+	+	+	Lp1
	13	-	Lp2-14	Lp12	+	+	+	-	-	Lp
	14	-	Lp2-14	Lp12	+	+	+	-	-	Lp
	15	-	Lp2-14	Lp12	+	+	+	-	-	Lp
	16	-	Lp2-14	Lp12	+	+	+	-	-	Lp
	17	-	Lp2-14	Lp12	+	+	+	-	-	Lp
	18	-	Lp2-14	Lp12	+	+	+	-	-	Lp
	19	-	Lp2-14	Lp12	+	+	+	-	-	Lp
	20	-	Lp2-14	Lp10	+	+	+	-	-	Lp
	21	-	Lp2-14	Lp12	+	+	+	-	-	Lp
	22	-	Lp1	Lp1	+	+	+	+	+	Lp1
	23	-	Lp2-14	Lp12	+	+	+	-	-	Lp
	24	-	Lp1	Lp1	+	+	+	+	+	Lp1
	25	-	Lp1	Lp1	+	+	+	+	+	Lp1
**LAXA**	21	-	Lp1	Lp1	+	+	+	+	+	Lp1
	22	-	Lp2-14	Lp10	+	+	+	-	-	Lp
	23	-	Lp2-14	Lp10	+	+	+	-	-	Lp
	53	-	NSR*	NSR*	-	+	-	-	-	non L
	54	-	NSR*	NSR*	-	+	-	-	-	non L
**Clinical strains**	Lp1 Lens	-	Lp1	Lp1	+	+	+	-	+	Lp1
	Lens *dotA*	-	Nd^§^	Nd^§^	+	+	+	-	+	Lp1
	Lp1 Paris	-	Lp1	Lp1	+	+	+	+	+	Lp1
	Lp1 Lorraine	-	Lp1	Lp1	+	+	+	+	+	Lp1
	Lp3	-	L	Lp3	Nd^§^	*+*	*+*	*-*	*-*	Lp
	Lp8	-	L	Lp6	Nd^§^	*+*	*+*	*-*	*-*	Lp
	*L. anisa*	++	L	*L. anisa*	*+*	*+*	*-*	*-*	*-*	L
	*L. taurinensis*	+	Nd^§^	Nd^§^	Nd^§^	*+*	Nd^§^	Nd^§^	Nd^§^	L
	*L. micdadei*	++	L	Nd^§^	Nd^§^	Nd^§^	Nd^§^	Nd^§^	Nd^§^	L
	*L.longbeachae*	+	L	*L. longbeachae*	*+*	*+*	*-*	*-*	*-*	L

* NSR: No Serotyping Reaction.

§ Nd: not determined.

### DNA analysis and molecular diversity of environmental *L. pneumophila* strains

**Figure 1:**
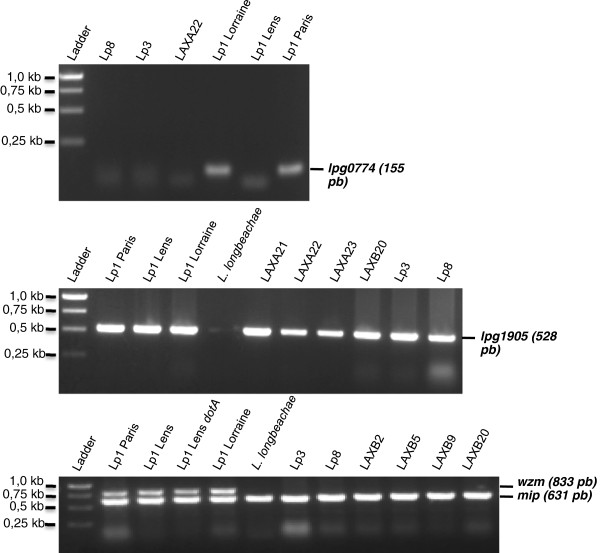
**Examples of PCR Amplification of several *Legionella pneumophila *genes: *lpg0774*, *lpg1905*,*wzm *and *mip*.** The ladder was the GeneRuler 1kb DNA ladder (Fermentas SM0311).

Molecular typing of the all environmental isolates allowed us to confirm the classification obtained by serotyping (Table 1). Actually, we used current standards in molecular diagnosis of the genus *Legionella: mip* gene (“Macrophage infectivity potentiator”), 16S rRNA genes [17]. Both genes were amplified by PCR from bacterial lysates of the 30 environmental isolates. Then, the discrimination of the specium *pneumophila* was performed by amplifying the gene *lpg0774*[18]. Finally, Lp1 typing of seven environmental *Legionellae* was obtained by independent gene amplifications of *lpg1905* and *wzm* (a gene belonging to the cluster coding for the lipopolysaccharide biosynthesis) [11,18]: LAXA21, LAXB6, LAXB8, LAXB12, LAXB22, LAB24 and LAXB25 (Table 1; Figure 1).

Thus, the LAXB environmental strains we isolated from the spring S mainly belong to Lp12 (15 isolates) and to a lesser extend to Lp1 (6 isolates) and Lp10 (3 isolates); it is interesting to underline that the isolate LAXB11 was classified as *L. pneumophila* only at the molecular level, and not by serotyping which could suggests a new serogroup. With regard to the LAXA strains, Lp10 (2 isolates) and Lp1 (1 isolate) were also identified, but Lp12 was not detected. Two isolates, LAXA53 and LAXA54, were classified as non *Legionella* species and were indeed further identified as *Mycobacterium* isolates on the basis of their 16S rRNA sequences using a different set of 16S rRNA primers (data not shown). The small number of Lp isolated in the LAXA campaign does not allow to draw any conclusion about the persistence of Lp between August and December 2010.

**Figure 2:**
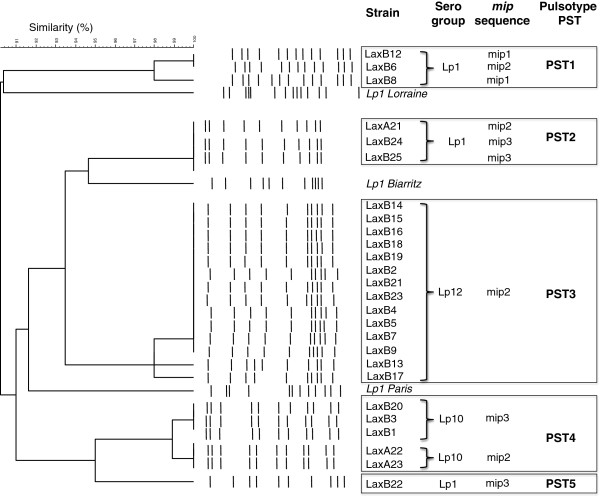
***Legionella pneumophila *typing.** The dendogramm represents the relationships of environmental and clinical strains of *Legionella pneumophila.* Patterns were generated by pulse field gel electrophoresis (PFGE) of total bacterial DNA and then clustered by unweighted pair group method with arithmetic averages algorithm.

In order to assess the molecular diversity, DNA of 26 LAXA and LAXB strains (7 Lp1, 5 Lp10 and 14 Lp12; LAXB10 strain did not grow anymore after a long term freezing period) was analyzed by PFGE and led to the identification of five main patterns (PST1 to PST5). It is clear that these five patterns are different from those of other known *L. pneumophila* clinical isolates as Lp1 strains Lorraine, Biarritz and Paris (see Additional file 1; Figure 2) but also Lp1 Lens, Philadelphia and Corby (data not shown). It is interesting to stress that Lp10 and Lp12 strains were grouped in two independent specific patterns (PST4 and PST3, respectively). By contrast, the 7 Lp1 strains were differentiated in three distinct patterns (PST1, PST2 and PST5) which suggest a genomic diversity.

**Figure 3:**
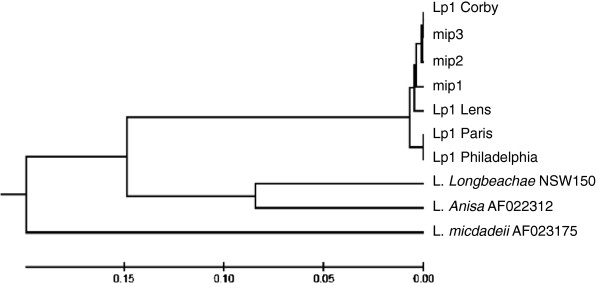
**Phylogenetic tree (Neighbor-joining) of *mip *sequences from *L. pneumophila *sg 1 clinical and environmental (*mip1, mip2 *and mip3) strains and *L. non-pneumophila *strains.**

In order to assess more finely this molecular diversity, the *mip* sequences of 27 *L. pneumophila* strains were determined and compared. All *mip* sequences were performed on both strands and no mismatch was identified. The 27 sequences comparison the led us to identify three different types of *mip* sequence, so-called *mip1*, *mip2* and *mip3*. These sequences exhibit a high identity (> 99%) and only differ by few substitutions (see Additional file 2): 5 substitutions between *mip1* and *mip2* sequences, 4 between *mip1* and *mip3* and a unique substitution between *mip2* and *mip3*. It must also be underlined that these three *mip* sequences are very close to those of known clinical isolates (identity > 99, 6%), and the *mip3* sequence is even completely identical to the *mip* sequence of the Lp1 clinical strain Corby (see Additional file 2). Actually, this sequence-based classification not only confirmed results obtained with other typing approaches (serotyping and molecular typing) but also allowed us to position the different environmental strains within the *specium pneumophila* (Table 2; Figure 3). Analyses of *mip* sequences confirmed the homogeneity of Lp12 strains belonging to the unique pulsotype PST3 and characterized by a unique *mip* sequence (*mip2*) (Table 2; Figure 2). Besides, this approach revealed a genetic diversity within the five Lp10 strains belonging to the pulsotype PST3 but differentiated by two *mip* sequences, *mip2* and *mip3*. Finally, a high genetic diversity was also observed within PST1 and PST2 pulsotypes, where the environmental Lp1 strains could be discriminated according to the three *mip* sequences (Table 2).

**Table 2:** **Classification of the 27 environmental *L. pneumophila *strains according to serogroup (sg), pulsotype (PST) and *mip *sequence**

**Class**	**Sg**	**PST**	***mip***	**Environmental isolates**	**Isolate number**	
**1**	sg1	PST1	*mip1*	LAXB8, LAXB12	2	7 Lp1
**2**	sg1	PST1	*mip2*	LAXB6	1	
**3**	sg1	PST2	*mip2*	LAXA21	1	
**4**	sg1	PST2	*mip3*	LAXB24, LAXB25	2	
**5**	sg1	PST5	*mip3*	LAXB22	1	
**6**	sg10	PST4	*mip2*	LAXA22, LAXA23	2	5 Lp10
**7**	sg10	PST4	*mip3*	LAXB1, LAXB3, LAXB20	3	
**8**	sg12	PST3	*mip2*	LAXB2, LAXB4, LAXB5, LAXB7, LAXB9, LAXB13, LAXB14, LAXB15, LAXB16, LAXB17, LAXB18, LAXB19, LAXB21, LAXB23, LAXB10*	15	15 Lp12
					27	27

*LAXB10 was positioned into the class 8 according to serotyping and *mip* sequence.

### Cytotoxicity to *Acanthamoeba castellani*

**Figure 4:**
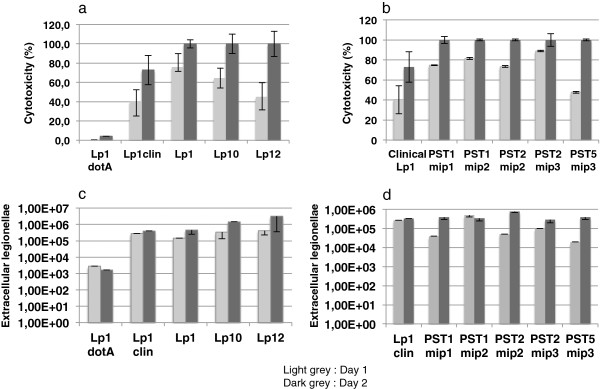
**Quantification of cytotoxicity and virulence of environmental *L. pneumophila *strains towards the amoeba *Acanthamoeba castellanii*.** Lp1 dotA : *dotA* mutant of Lp1 Lens; Lp1 clin: means of cytotoxicities (**a**) and virulences (**c**) of three clinical Lp1 strains (Lens, Paris and Lorraine). These means of cytotoxicities (**b**) and virulences (**d**) of three clinical Lp1 strains were compared to those of five independent pulsotypes (PST1 to PST5) of environmental Lp1 strains.

Alamar blue was used to quantify the viability of remaining amoeabae after *Legionella* infection (Figure 4a) and the cytotoxicity was assessed by percent of killed amoebae. The mean cytotoxicity of Lp1 clinical strains (Lens, Paris and Lorraine) was estimated to 40 and 73% after 24 h and 48 h post-infection, respectively. As expected, the avirulent mutant *dotA*, derived from the strain Lens [19] did not display any significant cytotoxicity (0 and 4% at 24 h and 48 h, respectively). Environmental strains isolated from the source S appeared much more cytotoxic than Lp1 clinical strains, especially at 48 post-infection: actually environmental Lp1, Lp10 and Lp12 are characterized by a cytotoxicity of 100% whatever their pulsotype (PST1, PST2 and PST5) or their *mip* sequence (*mip1, mip2 or mip3*) (Figures 4a and 4b).

### Virulence towards *Acanthamoeba castellani*

Lp1 clinical strains involved in LD outbreaks (Lens, Lorraine) and the worldwide epidemic and endemic strain Paris were used as virulent references. 1 × 10^5^ and 4, 5 × 10^5^ extracellular clinical Lp1 cells were present in 3 μl samples taken after a 24 h and 48 h period of *A. castellanii* infection, respectively (Figure 4c). In the same periods, legionella cells released from amoeba cells infected with the *dotA* mutant were 100-fold less numerous. Interestingly, the number of extracellular *Legionellae* cells resulting from amoeba infections with environmental strains was very close to that of clinical Lp1 with the exception of extracellular Lp12 strains associated with a 10-fold increase after a 48 h-period of infection. No significant difference of virulence was observed between the different classes of environmental Lp1 at 48 h post-infection, even if some strains appeared to present a weak delay of virulence at 24 h post-infection (Figure 4d).

A co-infection experiment was also conducted in *A. castellanii* with two representative strains of Lp1 (LAXB24) and Lp12 (LAXB2) environmental isolates. Duplex PCR analysis (using *wzm* and *lpg1905* primers) of extracellular bacteria revealed that 95% of 40 clones analyzed belonged to Lp12 strain (LAXB2), indicating the rapid and advantageous development of this Lp12 strain in competition to the Lp1 strain.

## Discussion

Our original approach of isolation of *L. pneumophila* cells from natural biofilms allowed to extend the knowledge of *Legionellae* populations contaminating a French Alpine thermal spa where several successive cases of LD occurred from 1986 to 1997. Other previous studies had reported the presence of five sg (1, 2, 3, 6 and 13) of free-living *L. pneumophila* isolated from water samples collected in different sites of the spa [12,13].

In contrast to these previous results, our work revealed that the sg 12 appears as the major population of *L. pneumophila* in biofilms developed within the spring S, a very original environment; besides, our results suggest that the 15 environmental Lp12 we isolated correspond probably to a unique strain; actually, all these Lp12 isolates could not differentiated at the DNA level (the same pulsotype PST3 the same *mip2* sequence) or at the level of cytotoxicity towards *Acanthamoeba castellanii*. All these data raise the hypothesis of a probable recently-emerged Lp12 strain with a capacity of rapid development in this specific environment, and more particularly within protozoa present in the spring S. This hypothesis is also supported by the co-infection experiment that pointed out the potential advantage of Lp12 strain in competition with Lp1 strain during amoeba infection. This probable emergence of Lp12 gives also an explanation to the absence of detection of Lp12 free-cells in water samples analyzed in other reports [12,13]. The absence of Lp12 from the LAXA strains we isolated in August 2010 could suggest an emergence of this strain in the spring S between the month of August and the month of December.

A similar hypothesis could be drawn for the sg 10, also absent from previous reports related to this thermal spa; the five Lp10 environmental isolates also characterized by a unique pulsotype (PST4); however, differences in two *mip* sequences (*mip2 and mip)* strongly suggests two Lp10 strains also recently appeared well-adapted in this site.

In contrast to Lp12 and Lp10, environmental Lp1 strains were already described in water samples collected from the three springs that fed the thermal spa. Unfortunately, Lp1 previously isolated from this thermal spa in 1988 and 1999 were no longer available; as a consequence, it is not possible to determine if the five classes of Lp1 we isolated result from a genetic evolution from a unique or several parental strain(s). Interestingly, the three distinct DNA patterns of environmental Lp1 were original and quite different of other known Lp1 clinical isolates involved in outbreaks. Besides, these environmental Lp1 were characterized by a higher toxicity and virulence towards amoebae than the Lp1 clinical isolates implied in outbreaks. At this stage, the possibility of a virulence decrease of Lp1 clinical isolates resulting from numerous times transfers in the laboratory cannot be ruled out. However, in our hands, no attenuation of virulence has been pointed out during the past 7 years. We can suppose that this high virulence of environmental isolates to amoebae is in relation with a long-term persistence of Lp1 probably in biofilms within the spring S. It is now recognized that the intracellular multiplication of Lp1 in amoebae enhanced their capacity of virulence towards alveolar human macrophages [20,21]. Taking into consideration their very high virulence capacity, these environmental Lp1 strains constitute good candidates for the recurrent LD observed in this French thermal spa. Moreover, the high virulence trait of Lp12 strains isolated in the spring S must also be taken into consideration. Indeed, a Lp12 strain has already been involved in a legionnaires disease in the past [22]. The whole-genome sequence of this clinical isolate Lp12 strain 570-CO-H has been recently characterized [23]. However, high virulence in amoebae does not completely correlate to high virulence in humans. Thus, higher virulence of environmental strains (Lp1, Lp10 and Lp12) compared to references Lp1 outbreaks strains does not absolutely mean higher risk of legionellosis. This hypothesis needs to be validated by further studies to assess the virulence of these environmental isolates towards human macrophages.

## Conclusion

This study highlights the role of mixed biofilms (protozoan and bacteria) of a site in the multiplication of virulent legionellae. Indeed, it has demonstrated the high virulence of environmental *Legionella pneumophila* serotype 1 isolates towards amoebae, a natural host in water spring; this is known to enhance *Legionella* virulence trait towards human macrophages. Moreover, it has shown the persistence capacity of *Legionella pneumophila* species in such an ecosystem. Finally, it also pointed out the biodiversity of *Legionella pneumophila* in their natural environment.

## Methods

### Environmental isolates

Glass slides were dipped into the contaminated spring S of a French Alpine thermal spa. After 15 days of incubation, the glass slides were covered with natural biofilms. These biofilms were harvested by scraping the glass slides and resuspended in 5 mL sterile water. Then, these suspensions were submitted to ultrasounds during 1 min in order to break up the aggregates formed by biofilms and to release bacterial cells. Bacterial suspensions were treated at 50°C during 30 min, and then submitted to an acidic treatment during 5 min by addition of 200 mM KCL/HCl pH 2.0. Aliquots (100 μL) were spread on agar GVPC medium (Oxoid, France) containing L-cysteine, iron pyrophosphate, ACES, charcoal and antibiotics (polymixin B, vancomycin, cicloheximide). After a 5 day-period incubation at 37°C, bacterial colonies with a fritted glass appearance were picked up and isolated again on GVPC. New independent colonies were picked up and suspended in cryotubes containing beads and bacterial preservers for storing at −20°C.

The *Acanthamoeba castellani* strain is an environmental isolate provided by F. Pernin (Institut des Sciences Pharmaceutiques et Biologiques - Faculté de pharmacie – Université Lyon 1, Lyon, France).

### Reference bacterial strains

Reference strains obtained from the National Centre of *Legionella* (Bron, France) were used as controls in different assays: *L. pneumophila* serogroup 1 (Lens, Paris, Lorraine), *L. pneumophila* ATCC 35096 (sg 8) and ATCC 33155 (sg 3), *L. anisa* G12108, *L. longbeachae* ATCC 35096, *L. micdadei* ATCC 33218 and *L. taurinensis* ATCC 700508. The *dotA* mutant is derived from the strain Lens and shows a severe defect of virulence and cytotoxicity [19]. Routinely, *Legionellae* were grown on buffered charcoal yeast extract (BCYE) agar (Oxoid, France) or in BYE liquid medium. *E. coli* DH5α was cultivated on Lysogeny Broth (LB) agar medium at 37°C and *Lactococcus lactis* subsp. *lactis* IL1403 was grown at 30°C on M17 agar medium [24].

### Serotyping of *Legionellae*

*Legionella* isolates were identified by polyclonal antisera coupled to latex-beads. Firstly, the Legionella latex test from Oxoid (DR0800M) allowed a separate identification of *Legionella pneumophila* serogroup 1 and serogroups 2–14, and the identification of seven non-*pneumophila* species: *L. longbeachae 1* and *2, L. bozemanii 1* and *2, L. dumoffii, L. gormanii, L. jordanis, L. micdadei and L. anisa*. Secondly, the 15 monovalent latex reagents prepared by bioMérieux allow the separate identification of 15 serogroups of *L. pneumophila* (bioMérieux, Craponne, France) [25]*.*

### *In situ* assay of catalase activity

The presence of bacterial catalase activity was detected using H_2_O_2_ as the substrate. A bacterial colony was picked up with a sterile loop and diluted into a 15 μL drop of 10% (vol:vol) H_2_O_2_, loaded on an empty Petri dish. The rapid formation (in a few seconds) of oxygen bubbles indicates a positive result. *E. coli* DH5α was used as the positive control (Cat^+^) and *Lactococcus lactis* IL1403 as the negative one (Cat^-^).

### Molecular identification and DNA amplification by PCR

Molecular markers used in this study were the following genes: *16S rRNA*, *mip*, *lpg1905*, *lpg0774* and *wzm* (Table 3). A soluble bacterial lysate containing the total DNA was prepared as following; a bacterial suspension was prepared in 40 μL of sterile water, treated at 90°C for 15 min, and centrifuged 13,000 rpm for 8 min. The supernatant corresponding to the bacterial lysate was kept and stored at −20°C.

**Table 3:** Couples of primers used in this study

**Gene**	**Primer name**	**Primer sequence**	**Amplicon size (pb)**	**Reference**
*16S RRNA*	Leg225	5^′^ AAGATTAGCCTGCGTCCGAT	654	[18]
	Leg858	5^′^ GTCAACTTATCGCGTTTGCT		
*mip*	mipLesnsens	5^′^ ATGAAGATGAAATTGGTGACTGCAG	607	[11]
	mipLensrev	5^′^ CAACGCTACGTGGGCCATA		
*lpg1905*	lpg1905sens	5^′^ TTGCCTAAAACTCACCACAGAA	528	[18]
	lpg1905rev	5^′^ ATGCCGCCCAAAATATACC		
*lpg0774*	lpg0774sens	5^′^ TGCTAACAACCACTATCCCAAA	155	[18]
	lpg0774rev	5^′^ GTTTCAATAAAAGCGTGCTCCT		
*wzm*	wzmsens	5^′^ ATGACCTCAATATCCTCAAAAACTCAG	833	[11]
	wzmrev	5^′^ TTATGCTCCATGTGATGAAATGC		

DNA amplification was performed with the 2 × PCR Master Mix DNAzyme II (Finnzymes) containing 0.04 U/μL DNAzyme™ II DNA polymerase, 400 μM of each dNTP, 3 mM MgCl_2_, 100 mM KCl and 20 mM Tris–HCl pH 8.8 (and stabilizers). The PCR mixture (25 μL) contained the 2 × PCR Master Mix DNAzyme II (12.5 μL), 10 mM forward and reverse appropriate primers (1.0 μL each) (Table 1), and the bacterial lysate (8.0 μL). Amplification of DNA was performed in a Ep-gradient Mastercycler (Eppendorf) at initial denaturation of 94°C for 2 min, followed by 35 cycles of 94°C for 1 min, 55°C for 1 min and 72°C for 1 min with a final extension at 72°C for 8 min. Reactions mixtures were then held at 10°C. 8 μL of the PCR amplification mixture was analyzed by gel electrophoresis in a 0.8% agarose gel stained with ethidium bromide (1.0 μg/mL) and photographed under U.V. transillumination.

### Purification and sequencing of PCR *mip* products

PCR *mip* products were analyzed by gel electrophoresis in a 0.8% agarose gel (50 mL) stained with 3 μL SYBR Safe DNA gel strain (Invitrogen). DNA products were visualized under blue U.V. transillumination and picked up with a band of agarose gel. Then PCR products were purified using GeneCleanR Turbo Kit (MP Biomedicals) according to the manufacturer’s instructions. Finally, the purified PCR products were suspended in 10 μL sterile water and then stored at −20°C. Sequencing was performed by GATC Biotech SARL (Mulhouse, France).

### PFGE subtyping

*Legionella* isolates were subtyped by pulsed field gel electrophoresis (PFGE) method as described previously [26]. Briefly, legionellae were treated with proteinase K (50 mg/mL) in TE buffer (10 mM Tris–HCl and 1 mM EDTA, pH 8) for 24 h at 55°C, and DNA was digested with 20 IU of SfiI restriction enzyme (Boehringer Mannheim, Meylan, France) for 16 h at 50°C. Fragments of DNA were separated in a 0.8% agarose gel prepared and run in 0.5× Tris-borate-EDTA buffer (pH 8.3) in a contour-clamped homogeneous field apparatus (CHEF DRII system; Bio-Rad, Ivry sur Seine, France) with a constant voltage of 150 V. Runs were carried out with increasing pulse times (2 to 25 s) at 10°C for 11 h and increasing pulse times (35 to 60 s) at 10°C for 9 h.

Then, the gels were stained for 30 min with a ethidium bomide solution and PFGE patterns were analyzed with GelComparII software (Applied Maths, Saint-Martens-Latem, Belgium).

### Quantification of *Legionella* virulence towards the amoeba *Acanthamoeba castellanii*

*Legionellae* were grown on BCYE agar and *A. castellanii* cells in PYG medium (Moffat and Tompkins, 1992) for five days at 30°C prior to infection. *A. castellanii* cells were first seeded in plates of 24 multiwell to a final concentration of 5 × 10^6^ cells per ml in PY medium (PYG without glucose. Plates were incubated during two hours at 30°C to allow amoeba adhesion. Then, *Legionellae* were added to an MOI (“multiplicity of infection”) of 5 (in duplicate). In order to induce the adhesion of bacterial cells to the monolayer of amoeba cells, plates were spun at 2000 × g for 10 min and incubated for 1 h at 30°C. Non-adherent bacteria were removed by four successive washings of PY medium. This point was considered as the initial point of infection (T0) and the plates were incubated at 30°C. Extracellular cultivable bacteria released from amoebae were quantified at 1 day and 2 days post-infection as follows. Aliquots (100 μL) of the supernatants were taken and diluted in sterile water to the final 10^-6^ dilution. Aliquots (3 μL) of the serial dilutions (10^-1^ to 10^-6^) were immediately spotted to the surface of agar BCYE plates. Independent bacterial colonies of serial dilutions were numbered after 5 days at 30°C.

In the co-infection experiment, the same cells amount of each strain was added to achieve a final MOI of 5. Extracellular bacteria (10^-5^ and 10^-6^ dilutions) were plated on BCYE agar, 48 h post-infection. Independent bacterial colonies were picked-up after 3 days at 30°C to perform a PCR analysis.

### Cytotoxicity to *Acanthamoeba castellani*

To quantify the viable *A. castellanii* cells remaining after infection with *Legionellae* (MOI 5), a monolayer of amoebae cells at the final concentration of 1 × 10^6^ cells per ml in a 96 multiwell plate was washed (fourfold) with PY and then treated with 10% Alamar blue (Invitrogen). Cytotoxicity of each *Legionella* strain was tested in triplicate. After an overnight incubation at 30°C, measurements were performed at the optical density (OD) of 570 nm and corrected for background at OD_600 nm_ with a μQuant microplate reader (Biotek Instruments Inc., Winooski, USA) The relative degree of amoeba mortality corresponds to the cytotoxicity and was expressed as the ratio of the OD value of infected monolayer to that of the uninfected one as following: [1-(mean OD value of infected/mean OD value of uninfected)] × 100%.

## Competing interests

The authors declare that they have no competing interests.

## Authors’ contribution

ZC isolated the environmental strains, performed serotyping, molecular typing, helped to perform cytotoxicity and virulence experiments and wrote portions of the final manuscript. FF and MR performed the PFGE analysis of the isolates. SJ planned PFGE experiments and helped in the preparation of the final manuscript. DA helped to plan molecular typing and performed cytotoxicity and virulence experiments in *Acanthamoeba castellani*, wrote large portions of the final manuscript. DF helped to plan isolation and first characterization of strains and helped in the preparation of the final manuscript. CG helped to plan molecular typing, performed cytotoxicity and virulence experiments in *Acanthamoeba castellani* and prepared the final manuscript. All authors read and approved the final manuscript.

## Supplementary Material

Additional file 1 **PFGE analysis of environmental and clinical *Legionella pneumophila *strains.*** Legionella* DNA samples were digested with SfiI restriction enzyme for 16 h at 50°C. Fragments of DNA were separated in a 0.8% agarose gel prepared and run in 0.5x Tris-borate-EDTA buffer (pH 8.3) in a contour-clamped homogeneous field apparatus with a constant voltage of 150 V. Runs were carried out with increasing pulse times (2 to 25 s) at 10°C for 11 h and increasing pulse times (35 to 60 s) at 10°C for 9 h. Click here for file

Additional file 2 **Multiple alignment of *mip *sequences from environmental (*mip1*, *mip2 *and *mip3*) and clinical *L. pneumophila* sg1 strains.** Clinical strains: Lp1Corby (NC009494.2), Lp1 Lens (NC006369.1), Lp1 Paris (NC006368) and Lp1 Philadelphia (AE017354.1). Click here for file
