# Supplementary material for: Molecular diversity and high virulence of Legionella pneumophila strains isolated from biofilms developed within a warm spring of a thermal spa
Source: BMC Microbiol. 2013 Jan 28;13:17. doi: 10.1186/1471-2180-13-17 (PMC3564684; doi:10.1186/1471-2180-13-17)

Additional file 1

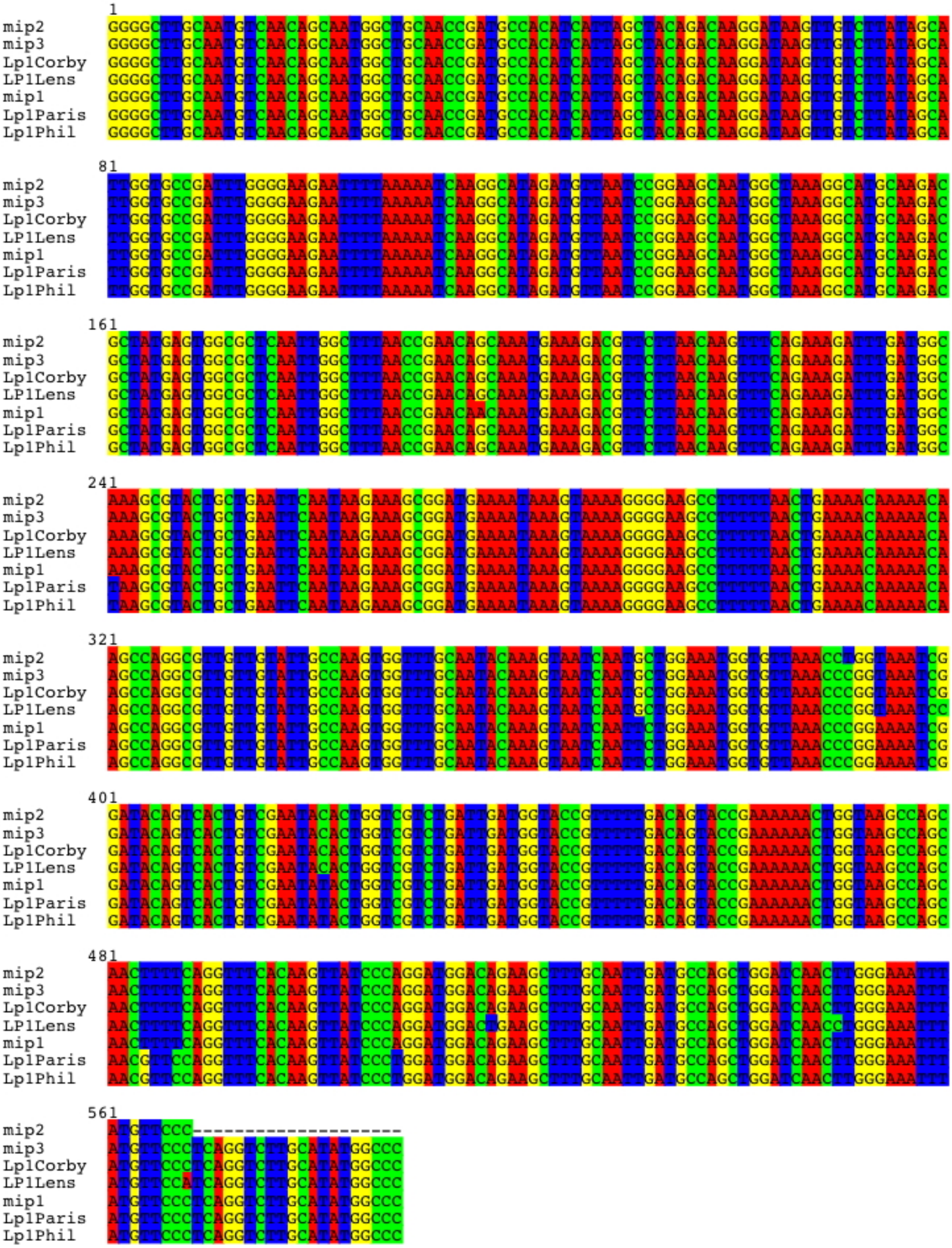

Supplement: Additional file 1 — PFGE analysis of environmental and clinical Legionella pneumophila strains. Legionella DNA samples were digested with SfiI restriction enzyme for 16 h at 50°C. Fragments of DNA were separated in a 0.8% agarose gel prepared and run in 0.5x Tris-borate-EDTA buffer (pH 8.3) in a contour-clamped homogeneous field apparatus with a constant voltage of 150 V. Runs were carried out with increasing pulse times (2 to 25 s) at 10°C for 11 h and increasing pulse times (35 to 60 s) at 10°C for 9 h. [file 1471-2180-13-17-S1.pdf]
