# Supplementary material for: Molecular diversity and high virulence of Legionella pneumophila strains isolated from biofilms developed within a warm spring of a thermal spa
Source: BMC Microbiol. 2013 Jan 28;13:17. doi: 10.1186/1471-2180-13-17 (PMC3564684; doi:10.1186/1471-2180-13-17)
Supplement: Additional file 2 — Multiple alignment of mip sequences from environmental (mip1, mip2 and mip3) and clinical L. pneumophila sg1 strains. Clinical strains: Lp1Corby (NC009494.2), Lp1 Lens (NC006369.1), Lp1 Paris (NC006368) and Lp1 Philadelphia (AE017354.1). [file 1471-2180-13-17-S2.pdf]

Additional file 2

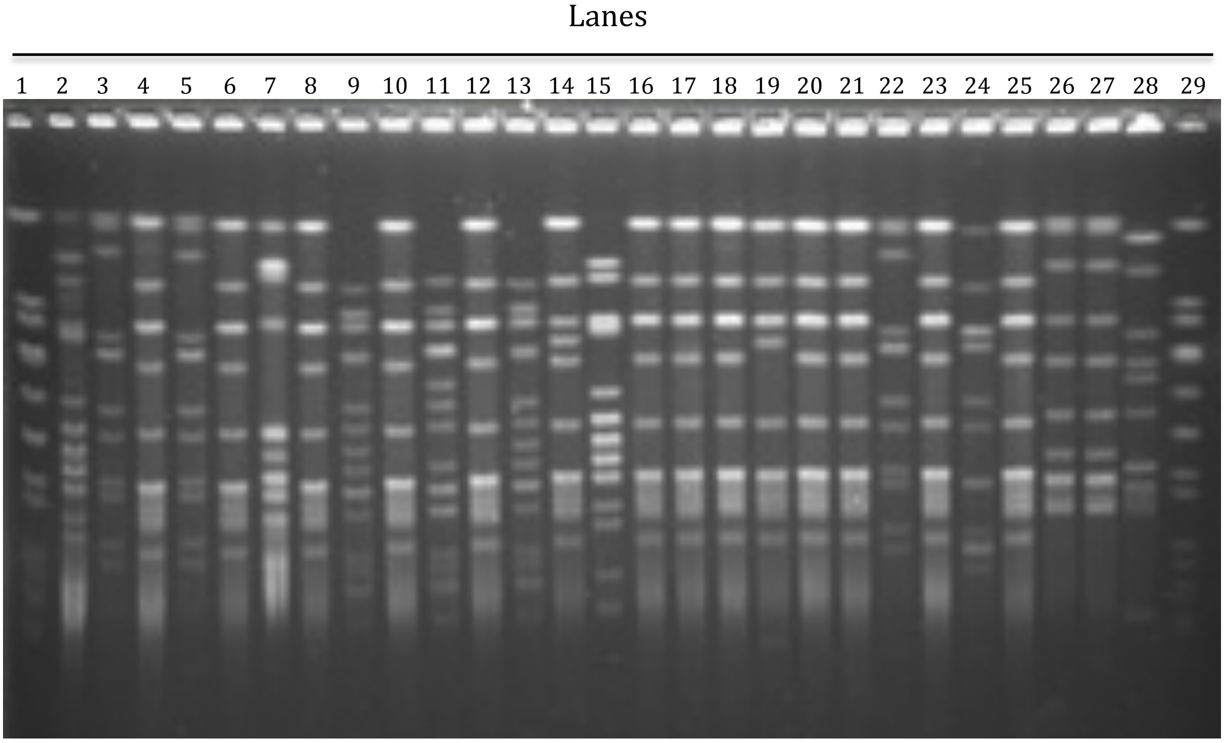

Lanes	Strains	Identification
1	*Staphylococcus aureus* NCTC 8325	
2	Lorraine strain	Lp 1
3	Lax B 1	Lp 10
4	Lax B 2	Lp 12
5	Lax B 3	Lp 10
6	Lax B 4	Lp 12
7	Paris strain	Lp 1
8	Lax B 5	Lp 12
9	Lax B 6	Lp 1
10	Lax B 7	Lp 12
11	Lax B 8	Lp 1
12	Lax B 9	Lp 12
13	Lax B 12	Lp 1
14	Lax B 13	Lp 12
15	Lorraine strain	Lp 1
16	Lax B 14	Lp 12
17	Lax B 15	Lp 12
18	Lax B 16	Lp 12
19	Lax B 17	Lp 12
20	Lax B 18	Lp 12
21	Lax B 19	Lp 12
22	Lax B 20	Lp 10
23	Lax B 21	Lp 12
24	Lax B 22	Lp 1
25	Lax B 23	Lp 12
26	Lax B 24	Lp 1
27	Lax B 25	Lp1
28	Biarritz strain	Lp1
29	Staphylococcus aureus NCTC 8325	
